# The Therapeutic Effect on Bone Mineral Formation from Biomimetic Zinc Containing Tricalcium Phosphate (ZnTCP) in Zinc-Deficient Osteoporotic Mice

**DOI:** 10.1371/journal.pone.0071821

**Published:** 2013-08-13

**Authors:** Joshua Chou, Jia Hao, Hirokazu Hatoyama, Besim Ben-Nissan, Bruce Milthorpe, Makoto Otsuka

**Affiliations:** 1 Faculty of Pharmacy, Research Institute of Pharmaceutical Science, Musashino University, Nishi-Tokyo, Japan; 2 Oral Implantalogy and Regenerative Dental Medicine, Tokyo Medical and Dental University, Tokyo, Japan; 3 Advanced Tissue Regeneration and Drug Delivery Group, Faculty of Science, University of Technology Sydney, Sydney, New South Wales, Australia; UC Davis School of Medicine, United States of America

## Abstract

The aim of this study was to evaluate the therapeutic efficacy of biomimetic zinc-containing tricalcium phosphate (ZnTCP) produced by hydrothermally converting calcium carbonate exoskeletons from *foraminifera*, in the treatment of osteoporotic mice. X-Ray powder diffraction showed crystallographic structures matching JCPDS profile for tricalcium phosphate. Mass spectroscopy used to calculate total composition amount showed similar amount of calcium (5×10^4^ µg/g) and phosphate (4×10^4 ^ppm) after conversion and the presence of zinc (5.18×10^3^ µg/g). *In vitro* zinc release showed no release in PBS buffer and <1% zinc release in 7 days. *In vivo* evaluation was done in ovariectomized mice by implanting the ZnTCP samples in the soft tissues near the right femur bone for four weeks. Thirty ddY mice (5 weeks old, average weight of 21 g) were divided into six experimental groups (normal, sham, OVX, β-TCP, ZnTCP and direct injection of zinc). CT images were taken every two weeks where the bone mineral density (BMD) and bone mineral content (BMC) were calculated by software based on CT images. The ZnTCP group exhibits cortical and cancellous bone growth of 45% and 20% respectively. While sham, OVX and β-TCP suffered from bone loss. A correlation was made between the significant body weight increase in ZnTCP with the significant increase in plasma zinc level compared with OVX. The presented results indicate that biomimetic ZnTCP were effective in preventing and treating bone loss in osteoporotic mice model.

## Introduction

The development of advanced and innovative drug delivery systems has over the years reached a paucity in which a biomimetic approach can offer new potentials and inspire future advancements in this area. Since the development of hydrothermal conversion of calcium carbonate from coral exoskeletons to calcium phosphate by [1], theses materials have been used successful in various bone augmentation applications. Our group has shown that fossilized *foraminifera*, available commercially, can be use as a drug carrier for antibiotics [2], bisphosphonate [3] and simvastatin [4].

*Foraminifera* possess a naturally uniform and interconnected porous network allowing consistent drug loading and release. Through hydrothermal conversion this unique structure can be preserve while transforming the material to tricalcium phosphate [5]. The present study seeks to combine a biomimetic material coupled with synthetic modifications to achieve a therapeutically relevant delivery system in the treatment of osteoporotic mice. Over the past years, significant interest has been vested on the modifications of biomaterials by incorporating inorganic dopants such as magnesium and strontium for there ability to stimulate osteoblasts while inhibiting osteoclasts and increasing mechanical properties of the bone [6]–[9]. While these have been used with degrees of success for various bone repair applications, one key biological element remains vastly overlooked in comparison. Zinc, a key element responsible in the regulation of cells, has shown to possess stimulatory effects on osteoblast bioactivity and bone formation [10]–[12]. In the past several years, different types of Zn-TCP formulations have been developed. Injectable Zn-TCP powders have shown to increase the BMD in osteoporotic rats [13], injectable Zn-TCP nanoparticles on jawbone bone mineral density have shown increased bone formation and mechanical strengths in osteoporotic rats [14] and in rabbit femora it was shown that over 50% new bone was formed [12]. More recently, studies have shown a relationship between osteoporosis and zinc deficiency in particularly in the elderlies [15]. However as a resorbable ceramic, TCP possess a higher solubility rate both *in vitro* and *in vivo*. If TCP degrades too rapidly, this may result in zinc ion released at high levels causing severe toxicity reactions [12], [16]. Furthermore, most Zn-TCP ceramic require sintering at high temperatures which can degrade the material’s mechanical properties and can be challenging for large-scale productions.

In this study, a new novel zinc delivery system is developed based on using *foraminifera* with low-temperature hydrothermal conversion to produce Zn-TCP which was than inserted into the soft tissues near the femur bone in a zinc-deficient osteoporotic mice model. While different systems of zinc containing tricalcium phosphate have been developed over the years, this study to the best knowledge of the authors is the first demonstration of using a biomimetic material as a carrier for zinc. This study will investigate if biomimetic Zn-TCP is able to induce beneficial pharmacological effect in treating osteoporotic mice.

## Materials and Methods

### Hydrothermal Synthesis and Characterization of Zn-TCP Material

**Figure 1:**
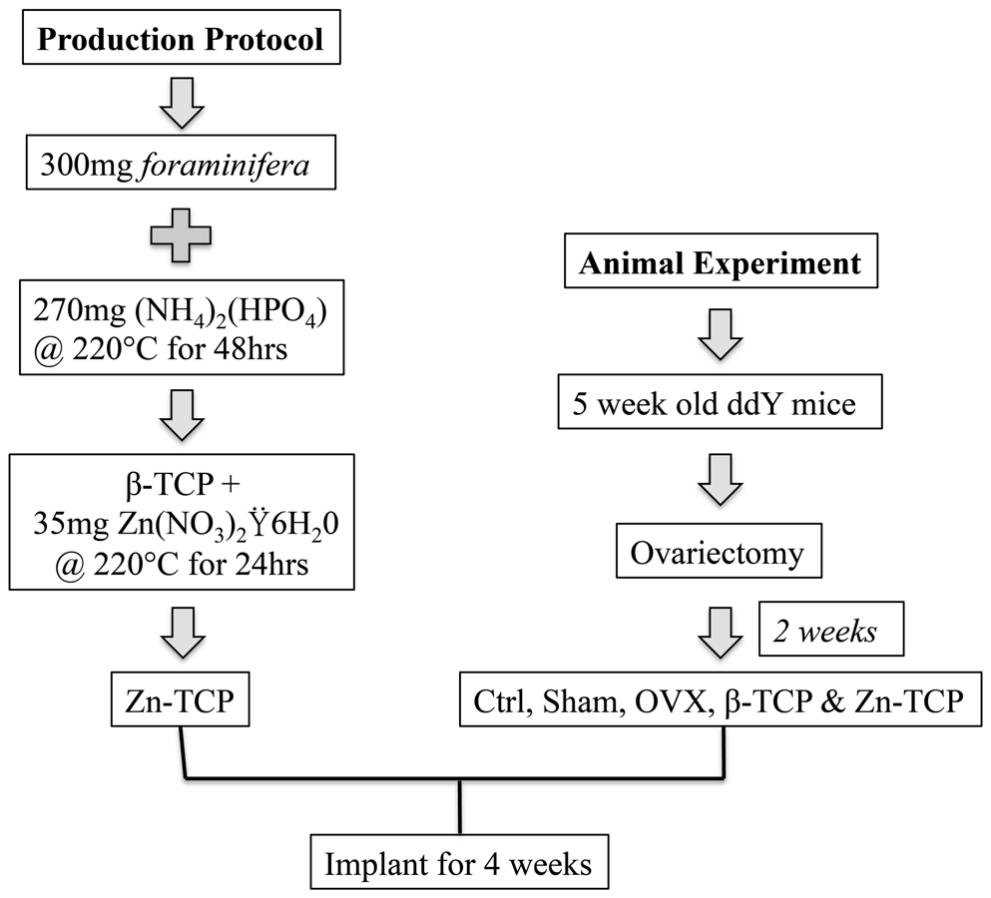
An outline of the experimental protocol for the production of Zn-TCP and procedure for the animal experiment.

Calcium carbonate samples from *foraminifera* were purchased commercially (Business Support Okinawa Co. Ltd., Japan) and no specific permits or licenses were required for the described study. The samples were cleansed in sodium hydrochlorite for 20 mins and dried at 40°C for 2 hours to remove any residual organics. The hydrothermal conversion was performed by immersing 300 mg of *foraminifera* with aqueous diammonium hydrogenphosphate [(NH_4_)_2_(HPO_4_)] (Wako Chemical Co., Tokyo, Japan), adjusted to yield Ca/P molar ratios of 1.5 to produce β-TCP and 30 mg of zinc nitrate hexahydrate (Zn(NO_3_)_2_⋅6H_2_0, Wako Chemical Co., Tokyo, Japan) and heated to 220°C for 48 hours in a pressure vessel. A schematic diagram of the experimental design is shown in Figure 1. Crystallographic profiles of the samples before and after hydrothermal conversion were measured by X-ray diffraction (XRD) (RINT- Ultima-III, Rigaku Co., Japan; CuKα radiation, 40 kV, 40 mA) and matched with JCPDS database. The surface morphological features of the Zn-TCP samples were observed by scanning electron microscopy (SEM) (JEOL JSM-7600F, Field Emission SEM, 10KV) before and after implantation. The excised samples were treated in 10% formaldehyde for 24 hours at 2°C and dehydrated in 60, 70, 80, 90 and 100% alcohol and allowed to dry before observing under SEM. For quantifying the chemical composition of the samples, approximately 0.005 g of sample was digested with 0.25 mL of HNO_3_. Once the digestion was completed the sample volume was made up to 5 mL. The samples underwent a further 1∶100 dilution with a 1% nitric acid solution before ICP-MS analysis. Samples were diluted further in nitric acid as required. An Agilent Technologies 7500ce series ICP-MS was used with sample introduction via a micromist concentric nebuliser (Glass expansion) and a Scott type double pass spray chamber cooled to 2°C. The sample solution and the spray chamber waste were carried with the aid of a peristaltic pump. The ICP operating parameters and the lens conditions were selected to maximise the sensitivity of a 1% HNO_3_:HCl solution containing 1 ng/ml of Li, Co, Y, Ce and Tl. Helium was added into the octopole reaction cell to reduce interferences. Calibration curves were constructed and the results analysed using Agilent Technologies Masshunter software. Mg, P, Ca, Zn and Sr stock solutions were obtained from Choice Analytical, Thornleigh, Australia. Baseline nitric acid (HNO_3_) was purchased from Seastar chemicals, Sidney, Canada. Calibration standards were prepared in 1% nitric acid. Samples were spiked with nitric acid to a level of 1% to matrix match the calibration standards.

### Measurement of *in vitro* Zinc Release

The zinc release rate was measured in phosphate buffer saline (PBS) and in acetate buffer (pH 4.5) prepared in accordance with Japanese Pharmacopeia. One ZnTCP sample was immersed in 10 mL buffer solution regulated thermostatically in a 37.0±0.1°C water bath shaken at 100 rpm. After every 24 hours for 7 days, the entire dissolution medium was collected and replaced with fresh buffer solution. Zinc concentration from the collected medium was measured by zinc detection kit (Metallo Assay Zinc LS, AKJ Global Technology Co. Ltd., Japan) at 560 nm using a UV-VIS spectrophotometer (Type UV160, Shimadzu Co., Japan).

### Animal Surgery and Procedures

Animal surgery and care was performed in accordance to the Animal Care Committee guidelines and approval from the Animal Ethics Committee at the Research Institute of Pharmaceutical Sciences, Musashino University. Thirty mice were assigned randomly into six groups (n = 5) as outlined in Table 1: 1) normal, 2) sham, 3) ovariectomized (OVX), 4) beta-tricalcium phosphate (β-TCP), 5) zinc-tricalcium phosphate (Zn-TCP) and 6) direct injection of zinc at approximately same concentration as Zn-TCP group (DI/ZN). Ovariectomy (OVX) was performed on thirty female ddY mice averaging 21 g in weight (Clea Co., Japan) under anaesthesia by intraperitoneal administration of sodium pentobarbital (0.1 mL/kg). During this time and for the duration of the experiment, groups 2–6 were fed a special diet (calcium, vitamin D and zinc deficient, Clea Co. Ltd, Japan) to induce and maintain the osteoporotic condition in the mice. Group1 (normal) received a normal diet without ovariectomy. Two weeks after ovariectomy, the mice were anesthetized by intraperitoneal administration of sodium pentobarbital (0.1 mL/kg) where five samples (15 mg) were implanted into the soft tissues near the right femur of the mice. After 4 weeks, the mice were euthanized by overdose of diethyl ether anesthesia, and the implanted samples and the femurs were excised for further analysis.

**Table 1:** Assignment of experimental groups and treatments.

Group	Diet	Type	Treatment	Group Acronym
1 (Control)	Normal food	Normal mice	None	Normal
2 (Control)	Special*	OVX mice	Sham	Sham
3 (Control)	Special*	OVX mice	Ovariectomized	OVX
4	Special*	OVX mice	β-TCP material implanted	β-TCP
5	Special*	OVX mice	Zn-TCP material implanted	Zn-TCP
6	Special*	OVX mice	Direct injection of equal amount of zinc to Zn-TCP	DI/ZN

* Special diet refers to calcium, vitamin D, magnesium and zinc deficient diet.

### *In vivo* Analysis

Bone mineral density (BMD) and bone mineral content (BMC) were calculated by X-Ray CT (LCT-200A, ALOKA, Japan) images and analyze in system software (Latheta, ALOKA, Japan) based on CT images taken every two weeks. The measurement of zinc concentration was based on blood plasma collected after the mice were euthanized after four-weeks. Zinc concentration in the plasma was determined by a zinc detection kit (Metallo Assay Zinc LS, AKJ Global Technology Co. Ltd., Japan) according to manufacturer’s protocols and measuring at 560 nm using a UV-VIS spectrophotometer (Type UV160, Shimadzu Co., Japan).

### Statistical Analysis

All data were examined based on five different measurement values and presented as mean ± standard deviation. Repeated measurement analysis of variance (ANOVA) was used to determine significant differences among the groups and a p-value <0.05 was considered significant.

## Results

### Characterization of Zn-TCP Delivery System

**Figure 2:**
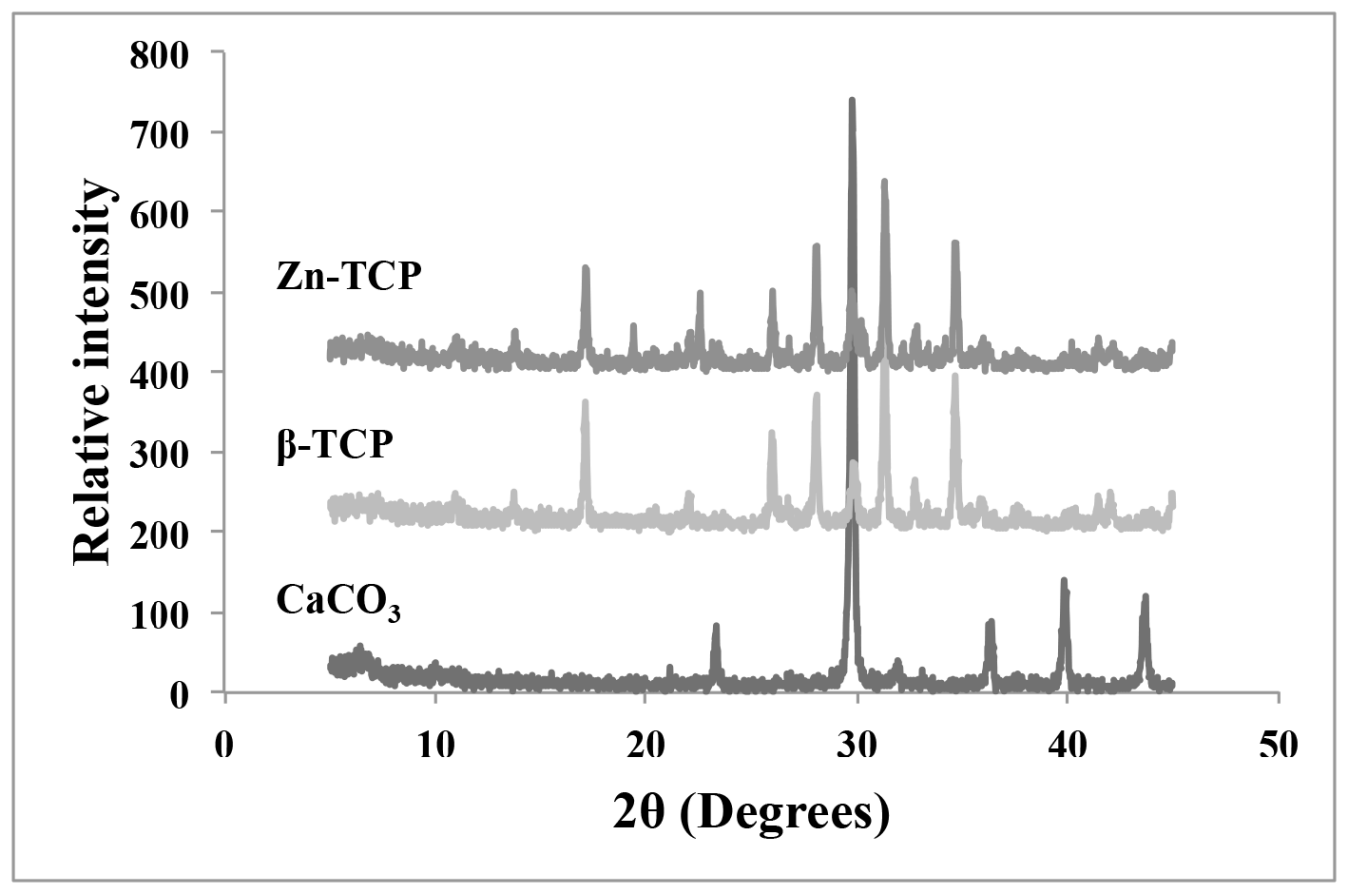
The XRD pattern corresponding to *foraminifera,* β-TCP and Zn-TCP showing peaks matching calcium carbonate (JCPDS 5-0586) and tricalcium phosphate (JCPDS 9-169).

Zn-TCP material was produced by hydrothermally converting calcium carbonate from *foraminifera*. A more detailed physico-chemical characterization was performed in a previous study [17]. Figure 2 shows the X-ray powder diffraction profile of the *foraminifera,* β-TCP and Zn-TCP samples. The diffraction patterns match JCPDS 5-0586 for calcium carbonate and JCPDS 9-169 peaks corresponding to β-TCP. There was no significant difference with the Zn-TCP peak patterns, which suggest that the zinc was successfully incorporated by substituting in place for calcium. A detailed overview of the compositional amount of calcium, phosphate and zinc from the samples are outlined in Table 2. The amount of calcium in β-TCP and Zn-TCP was calculated to be 4.45×10^4^ and 5.10×10^4^ µg/g and phosphate, 4.10×10^4^ and 3.75×10^4^ µg/g respectively. The β-TCP sample contained no amount of zinc while Zn-TCP had 5.18×10^3^ µg/g, which confirms that the conversion process was successful.

**Table 2:** Chemical composition of sample material.

	Calcium	Phosphate	Zinc
β-TCP	**4.45×10^4^**±1.2×10^3^	**4.1×10^4^**±5.3×10^3^	0
Zn-TCP	**5×10^4^**±4.7×10^3^	**3.75×10^4^**±1.4×10^3^	**5.18×10^3^**±3×10^2^

### Evaluation of *in vitro* Release of Zinc

**Figure 3:**
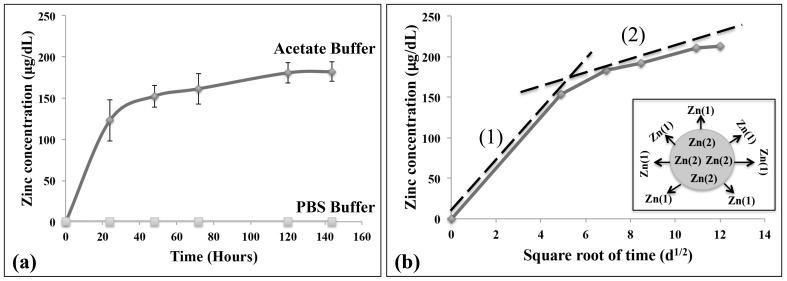
The *in vitro* (a) zinc release in acetate buffer (70%) and PBS (0%) over 7 days and (b) the complementing Higuchi plot showing two phases of zinc release: surface zinc release (slope 1) and drug diffusion release from the matrix material (slope 2).

The release of zinc from Zn-TCP in acetate and PBS buffer are shown in Figure 3 (a). There was an initial burst release of zinc during the first 24 hours in acetate buffer solution before the release reached a constant concentration around 150–160 µg/dL. Taking into account the average zinc concentration in each Zn-TCP sample, this only accounts for <1% of the total zinc composition. This suggests that the majority of zinc are still within the material. No zinc release was observed in the PBS buffer solution. The zinc release rate was rapid initially in acetate buffer, suggesting that zinc was first released from the surface as the material slowly degrades and subsequent release through the macro- and micro pores. Therefore the zinc release from porous calcium phosphate matrix system would follow the Higuchi equation [18]:
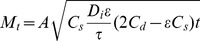
(1)where *M_t_* is the amount of drug release after time *t*, *A* is the matrix surface area, *D* is the diffusion coefficient of the drug, *C_s_* is the solubility, *C_d_* is the concentration of the drug in the matrix, *τ* is the tortuosity and *ε* is the porosity of the matrix. The *in vitro* zinc release profile in acetate buffer was plotted with zinc concentration against the square root of time as shown in Figure 3 (b). The plot shows two phases of zinc release. The initial burst release was due to conventional release of zinc from the surface of the ZnTCP as illustrated by slope (1). The second order release profile shows a linear slope (2) in the Higuchi plot suggesting that the release of zinc from the matrix material is based on a drug diffusion process through the macro- and micro pores of ZnTCP. The release rate constants based on the two slopes of the plots were calculated to be: slope (1) 25 µg/d^1/2^ and slope (2) 6 µg/d^1/2^.

### Examination of Zn-TCP Structural Morphology

**Figure 4:**
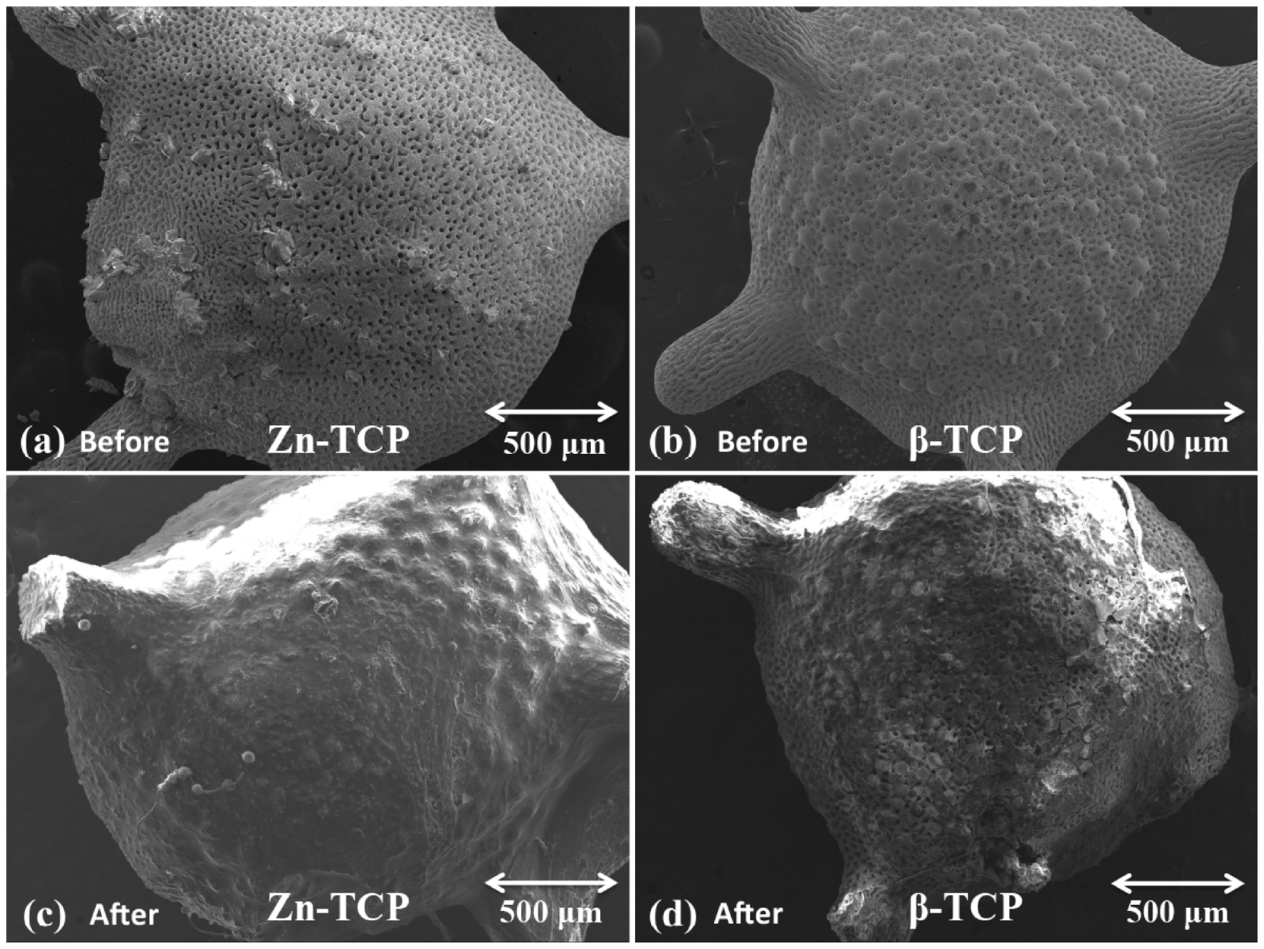
SEM images showing the morphological structure of Zn-TCP (a) before, (c) after and β-TCP (b) before, (d) after 4 week implantation in OVX mice. From the images there is no significant structural change to the surface morphology with the exception of tissue growth around the sample material.

Figure 4 shows SEM images of the structural morphology of β-TCP [(b),(d)] and Zn-TCP [(a),(c)] before and after implantation in OVX. It can be seen from the figures that after 4 weeks the structure of both samples remained unchanged except for the growth of fibroblastic tissues around both the β-TCP and Zn-TCP sample (c). This indicates that the amount of zinc released is not detrimental to the surrounding tissues.

### Effect of Zn-TCP and Direct Injection of Zinc on Osteoporotic Mice

**Figure 5:**
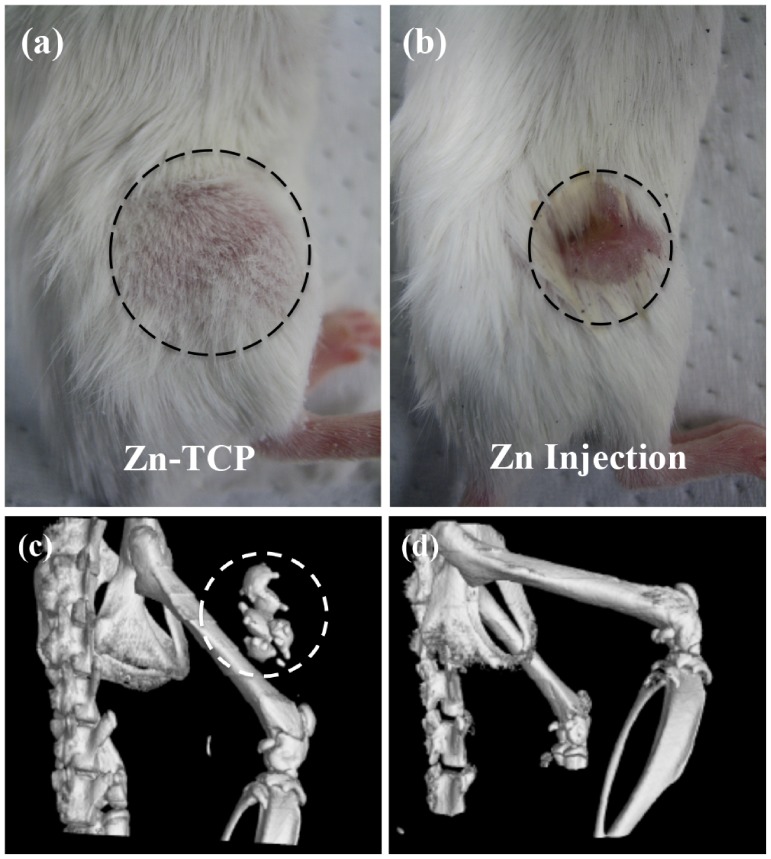
Image showing the difference from macroscopic observation between (a) implanted Zn-TCP and (b) direct injection of zinc exhibiting severe muscle deterioration. Computer reconstruction of the femur bone showed (c) the Zn-TCP placed near the femur bone and (d) the inhibition of bone extension due to the muscle breakdown.

Direct injection of zinc (DI/ZN), at approximately the same concentration (20 µg/week) as Zn-TCP, was injected in the soft tissues near the right femur of the osteoporotic mice as a way of comparing the two-treatment method. Unfortunately, all the mice in the DI/ZN group did not survive past week 2 and as such no analysis was performed for this group in the subsequent analysis. Observation of the mice (Figure 5) in (a) Zn-TCP and (b) DI/ZN group showed significant deterioration of the soft tissue at the zinc injection site of the DI/ZN group. This rendered the mice the inability to possess normal function of its right leg. Using the CT images, a complete 3D reconstruction of the femur was made to observe the position of the Zn-TCP samples in reference to the femur bone. Figure 5 (c) show that the Zn-TCP was indeed placed near the femur bone and Figure 5 (d) showed that how the femur bone was pushed together from the soft tissue deterioration. This serious side effect associated with highly localized zinc concentration was not observed in the Zn-TCP group.

### Effect of B-TCP and Zn-TCP on Mice Body Weight

**Figure 6:**
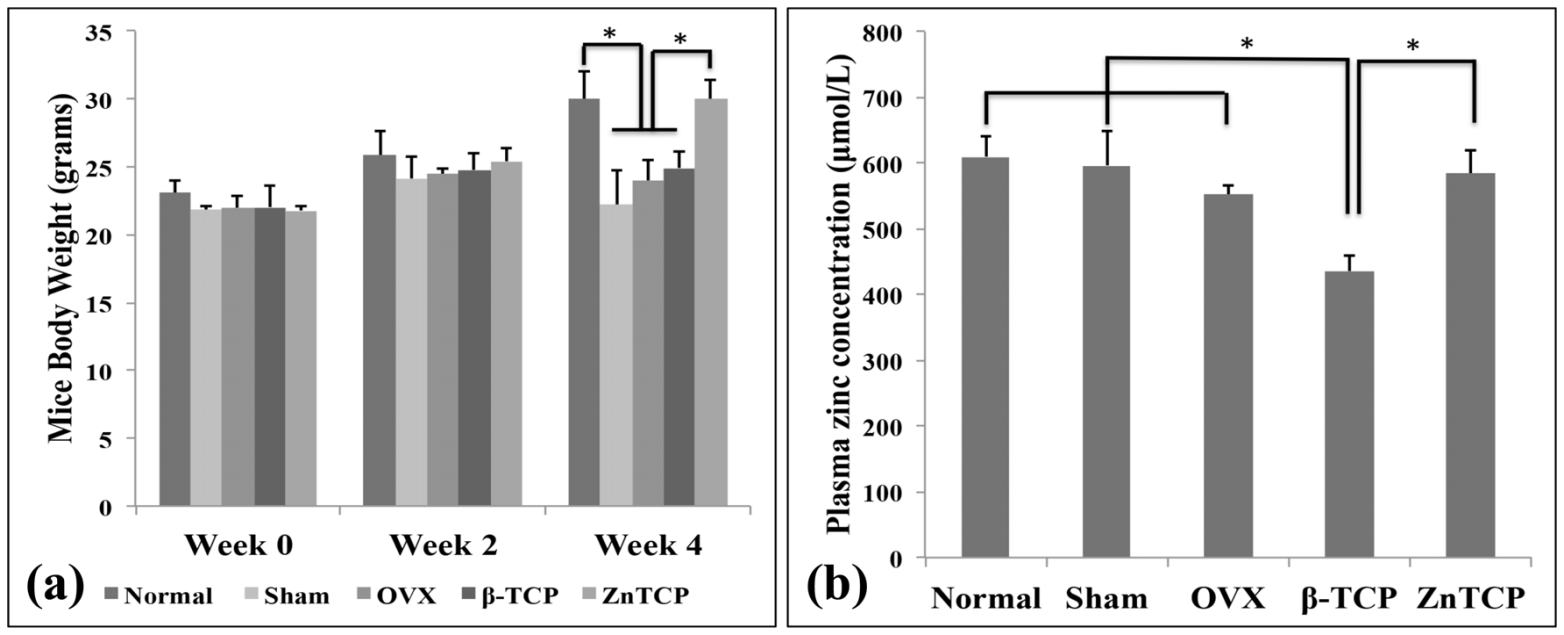
From these results a close relationship between the (a) the mice body weight and (b) plasma zinc level was observed. Zn-TCP group showed a significantly higher body weight with increased plasma zinc level.

The body weights of the mice were monitored and recorded as this directly correlates to the amount of bone and soft tissues from the mice. The application of Zn-TCP to the osteoporotic mice led to a continuous and significant increase in body weight after 4 weeks to the same level as normal healthy mice [Figure 6 (a)]. In contrast, no significant increase in body weight was observed for the mice in sham, OVX and β-TCP groups.

### Effect of Zn-TCP on Plasma Zn Level of Osteoporotic Mice

Plasma zinc levels collected from the osteoporotic mice after 4-weeks [Figure 6 (b)] showed decreasing level of zinc concentration in the order of normal, sham, OVX, β-TCP and Zn-TCP. There was a significant decrease in zinc concentration between normal, sham and OVX compared with β-TCP and Zn-TCP. It should be noted that the mice were fed with a zinc deficient diet to observe the changes in the plasma zinc level.

### Effect of Zn-TCP on BMC and BMD

**Figure 7:**
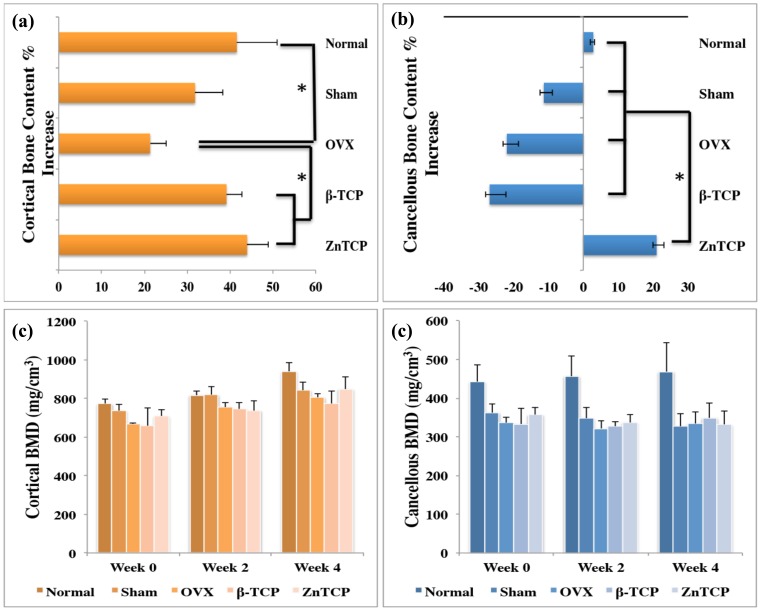
Based on the CT scans, the cortical and cancellous bone BMC and BMD were measured. The percentage difference in the BMC of (a) cortical bone showed consistent bone increase and (b) cancellous bone showing significant increase by Zn-TCP group. The BMDs of (c) cortical bone showed no significant changes while the (d) cancellous bone BMD showed only the normal group having a significant difference.

The percentage increase/decrease in the right femur cortical and cancellous BMC is shown in Figure 7 (a) and (b) respectively. β-TCP and Zn-TCP groups showed 40 and 45% increase respectively in cortical bone mass, which is significantly different compared with the OVX control group. The BMC of the cancellous bone displayed significant changes between the experimental groups. Sham, OVX and β-TCP exhibited 12, 22 and 30% reduction in cancellous BMC over the 4-week period while normal and Zn-TCP groups had 5 and 20% increase during the same period. Zn-TCP showed significantly higher increase in cancellous BMC compared with all experimental groups. Figure 7 (c) and (d) show the right femur BMD of the cortical and cancellous bone. The BMD of the cortical bone did not show any significant changes while the BMD from the cancellous bone of the normal group displayed significant increase with all the experimental groups during week 2 and week 4. The cancellous bone BMD from sham, OVX, β-TCP and Zn-TCP group remained constant during the experimental period.

## Discussion

In this study, the effect of biomimetic zinc containing tricalcium phosphate (Zn-TCP) on the bone mineral content of the femur bone was investigated in a zinc-deficient osteoporotic mice model. This model was selected as the mice are at a young age and still growing allowing the observation on the significance of zinc in bone growth under osteoporotic conditions. Furthermore, Zn-TCP and β-TCP were both implanted into the soft tissues near the right femur of the osteoporotic mice. This surgical insertion was made as a way to observe the localized therapeutic efficacy of Zn-TCP and does not reflect on the clinical application of this system. For clinical application, it is envision that the Zn-TCP could be implanted near the peritoneal cavity as a more optimal implantation site, which would reduce the impact on the patient’s quality of life. Zn-TCP samples were produced by hydrothermally converting calcium carbonate exoskeletons from *foraminifera*. This allows the conversion to tricalcium phosphate while preservation of the unique architectural structure of the exoskeletons. *Foraminifera* are abundant as fossils for the last 540 million years and can be found in all marine environments but different species exists depending on the local habitat. *Foraminifera* are single-celled organisms with shells consisting of multi-layer inner chambers commonly divided and added during its growth, which generate the distinct porous chambers. The novelty in using *foraminifera* as a carrier material is due to the interconnectivity of the uniform pores the material naturally possess allowing a more consistent drug loading and therefore a more predictable release rate. From the XRD results, the crystallographic structure of Zn-TCP is closely identical to β-TCP and from previous *in vitro* studies the zinc ions are incorporated in place of the calcium ions [17]. SEM images before implanting into the OVX mice also show zinc particles embedded on the surface of Zn-TCP and the overall structure remained structurally intact after being implanted for 4 weeks. Since the zinc are incorporated into the lattice structure and on the surface of the material, from the SEM images it is clear that the pores are unfilled allowing the infiltration of cells and blood once inside the body. Furthermore, this allows the extended option of incorporating and loading the carrier material with other stimulatory compounds that can address other therapeutic response and needs. With any drug delivery system, its therapeutic efficiency is highly dependent on the carrier’s ability to retain and release the loaded pharmaceutical compounds. *In vitro* zinc release in PBS buffer, which is calcium deficient and therefore simulates a similar condition to osteoporosis, showed no zinc release in 7 days. This suggests that the zinc incorporated in the TCP is relatively stable and can provide a long-term release of zinc. In contrast, Zn-TCP in acetate buffer (pH 4.5), which simulates an acidic environment commonly associated with localized inflammatory reaction after surgical incision, showed 70% zinc release in the same 7-day period. This shows that zinc is relatively stable and even in continuous acidic environment, zinc can still be retained in the ZnTCP material. The biological environment will notably be different and it is not expected that zinc from ZnTCP degradation will be this fast as evident by the excised samples. Since Zn-TCP has a low solubility rate, the release of zinc from calcium phosphate material would be in a well-controlled and slow manner [19]. *In vitro* zinc release was observed for 7 days as this provides a general overview of the zinc release pattern and any extended release studies would not correlate to the complex *in vivo* biological setting. After the samples were implanted, macroscopic examination of the mice revealed no inflammatory reaction to the implanted Zn-TCP. However serious muscle deterioration and inflammatory response was observed in the zinc injected group, which reached a toxic level after 2 weeks at which all the mice died as a result. This is a classic example showing how a high-localized concentration of pharmaceutics can elicit serious side effects while a controlled and slow release of the same compound can avoid such events. In this case, Zn-TCP delivery system is capable of preventing the associated side effects of high zinc concentration in the immediate area, which also reflects on the materials biological tolerance. In the present study, the body weight of the mice were observed during the experimental period as the differences in increase body weight can be attributed to the pharmacological effect of Zn-TCP. The weight of bones and muscles can account for approximately 20 to 50% of the total body weight, which can be use as a parameter that reflects on the bone generated. The body weight from the normal and Zn-TCP group increased significantly compared with sham, OVX and β-TCP after 4 weeks. As such, the significant weight gained by the Zn-TCP group can be attributed to the pharmacological effects of zinc released. A close correlation can be made with the significantly higher plasma zinc level found in the Zn-TCP group and the significant body weight in the mice. Conversely, a lower plasma zinc level in the β-TCP group was matched with no increase in body weight. Interestingly the plasma zinc level in the OVX group is higher compared with β-TCP. A hypothesis can be made that initially the plasma zinc level in the OVX mice was low and to compensate the zinc level, additional zinc was released from the bone to make-up for the zinc deficiency. While this would increase the zinc level, it will at the same time further deteriorate the conditions of the osteoporotic bone. β-TCP samples would release calcium as the material degrades therefore supplementing the body system with calcium which might explain why zinc plasma level was lower in this group. The results here further indicate that a sufficient level of calcium and zinc in the blood plasma is necessary for body growth. This suggests that lower zinc levels deactivate key enzymes essential for growth [20]. The BMD level of the cortical and cancellous bones reflects on the balance between absorption and deposition of bone minerals which the results showed that only the normal group had a significantly higher BMD compared with sham, OVX, β-TCP and Zn-TCP group. The BMDs from these groups remained unchanged during 4 weeks, which would suggest the rate of absorption and deposition remained consistent indicating that the effect of Zn-TCP was not sufficient enough to increase the BMD of cortical and cancellous bone of the OVX mice to the same level as the normal healthy mice group. In contrast, the BMCs of the femurs were measured to evaluate the local therapeutic effect of Zn-TCP. A plot of the BMC percentage increase over 4 weeks showed significant cortical bone growth from normal, β-TCP and Zn-TCP compared with OVX group. Without the continual stimulatory effect on bone formation, the osteoporotic condition will continue to resorb the cancellous bone. Most interestingly and significant indication that Zn-TCP is pharmacologically effective is the significant increase in the percentage of cancellous bone growth in 4 weeks. Here it shows that Zn-TCP was able to induce approximately 20% cancellous bone growth compared with the 5% growth in normal mice. This is as expected, since the normal group would have minimal growth as the mice matures. Sham, OVX and β-TCP group all showed decreased cancellous bone growth in the 4-week period, a trait consistent with the OVX condition. The presented results indicate that ZnTCP is capable of inducing localized cortical and cancellous bone growth in osteoporotic mice and show potentials for further investigation in larger animal models.

### Conclusion

This study shows that Zn-TCP produced by hydrothermally converting *foraminifera* is able to release zinc in a controlled and slow manner over an extended period of time. The results indicate that the Zn-TCP system is effective for localized therapy and that long-term sustained zinc release from Zn-TCP may further improve bone mineral density in Zn-deficient osteoporotic mice. Based on these *in vivo* results, it can be conclude that the Zn-TCP system is adequate and is therapeutically effective in inducing localized cortical and cancellous bone growth while promoting healthy body growth in osteoporotic mice.
